# Drug Use After Emergency Department‐Initiated Injectable Buprenorphine: A Secondary Analysis of the ED‐INNOVATION Ancillary Safety and Feasibility Trial

**DOI:** 10.1111/acem.70191

**Published:** 2025-11-24

**Authors:** Ethan Cowan, Gail D'Onofrio, Jeanmarie Perrone, Erik Anderson, James Dziura, Kathryn Hawk, Andrew Herring, Ryan McCormack, Manali Phadke, Elizabeth A. Samuels, David A. Fiellin

**Affiliations:** ^1^ Department of Emergency Medicine Rutgers New Jersey Medical School Newark New Jersey USA; ^2^ Department of Emergency Medicine Yale School of Medicine New Haven Connecticut USA; ^3^ Department of Medicine Yale School of Medicine New Haven Connecticut USA; ^4^ Department of Health Policy and Management Yale School of Public Health New Haven Connecticut USA; ^5^ Department of Emergency Medicine Perelman School of Medicine at the University of Pennsylvania Philadelphia Pennsylvania USA; ^6^ Department of Emergency Medicine Highland General Hospital‐Alameda Health System Oakland California USA; ^7^ Yale Center for Analytical Sciences (YCAS) Yale School of Public Health New Haven Connecticut USA; ^8^ Department of Epidemiology and Chronic Disease School of Public Health, Yale University New Haven Connecticut USA; ^9^ Ronald O. Perelman Department of Emergency Medicine New York University Grossman School of Medicine New York New York USA; ^10^ Department of Emergency Medicine, David Geffen School of Medicine University of California Los Angeles California USA

## Abstract

**Study Objective:**

To characterize opioid and nonopioid drug use in the week following emergency department (ED)‐initiated extended‐release buprenorphine (XR‐BUP) treatment using both self‐reported data and urine drug screens (UDS).

**Methods:**

This study uses data collected during a nonrandomized clinical trial of patients with untreated opioid use disorder (OUD), testing the safety and feasibility of initiating XR‐BUP in patients presenting with minimal to mild withdrawal. The study was conducted from July 2020 to May 2023 across four urban academic EDs in the Northeast, Mid‐Atlantic, and Pacific regions of the United States. A total of 100 participants, 18 years or older with OUD defined by DSM‐5 criteria, a clinical opiate withdrawal scale (COWS < 8), and a positive opioid urine screen were included. Individuals with recent MOUD treatment, presentation for overdose, or concurrent methadone use were excluded. All participants received a single subcutaneous injection of 24 mg XR‐BUP (CAM2038) during their ED visit. The primary outcomes were self‐reported daily opioid and nonopioid drug use over 7 days postinjection using daily Qualtrics surveys and UDS results on day 7.

**Results:**

Among participants who received XR‐BUP and completed daily surveys, 98% reported at least one opioid‐free day, and 63% reported no opioid use across all 7 days. Day 7 UDS results showed decreased detection of opioids, stimulants, and benzodiazepines. Reported polysubstance use also declined over the observation period.

**Conclusions:**

ED‐initiated XR‐BUP was associated with substantial reductions in opioid and polysubstance use during the first week post‐discharge, supporting its role in early overdose risk mitigation and highlighting its value as an ED‐based intervention for opioid use disorder.

**Trial Registration:**

ClinicalTrials.gov Identifier: NCT03658642

## 1 Introduction

Despite a recent decrease in opioid overdose deaths in the United States, the opioid epidemic remains a significant public health problem, with 54,000 opioid deaths reported in 2024 [1]. Emergency departments (EDs) have become key intervention points for individuals with opioid use disorder (OUD), providing an opportunity to initiate treatment and reduce overdose risk [2]. Multiple studies have demonstrated that ED‐initiated buprenorphine significantly increases the likelihood of patients engaging in continued addiction treatment after discharge [3, 4, 5, 6, 7]. However, little is known about patient experiences and substance use patterns in the period between ED‐initiated buprenorphine treatment and their first follow‐up appointment. This early posttreatment phase is particularly important, as factors such as continued drug use may elevate overdose risk and decrease the likelihood of treatment engagement [8, 9].

The first weeks following MOUD initiation are particularly high‐risk for morbidity and mortality [10, 11]. Studies indicate that within a year of an ED visit for OUD or a nonfatal opioid overdose, approximately 5.5% of patients die, with over 20% of these deaths occurring within the first month [12]. Initiating medications for opioid use disorder (MOUD), such as buprenorphine, during this period has been shown to reduce mortality by up to 50%, underscoring the importance of timely intervention [13]. However, while the long‐term benefits of MOUD are well established, little is known about how patients continue or modify their substance use in the days immediately following treatment initiation.

Further complicating this issue is the increasing prevalence of polysubstance use, including use of opioids with stimulants and/or benzodiazepines, which has been linked to higher overdose risk, lower rates of MOUD engagement, and poorer treatment retention [14, 15, 16]. Understanding drug use patterns during the early posttreatment phase is essential, as continued or escalating substance use can impact treatment retention and other clinical outcomes. However, measurement of drug use post‐ED discharge remains challenging. Existing ED‐based studies of post‐discharge drug use have relied primarily on self‐reported data, which may be subject to recall bias and social desirability effects [17]. The concordance between self‐reported drug use and objective measures, such as urine drug screens (UDS), remains poorly understood in this population and is complicated by time windows of detection of drugs and their metabolites [18].

This study sought to address these gaps by characterizing drug use patterns during the first week following ED‐initiated treatment with extended‐release injectable buprenorphine (XR‐BUP). By integrating both self‐reported and UDS data, this study aimed to provide a more comprehensive understanding of early posttreatment reduction in substance use. Identifying patterns of continued drug use in the immediate posttreatment period can help inform discharge counseling, harm reduction strategies, transition planning, and follow‐up care.

Specifically, our primary objective was to characterize drug use patterns in patients with untreated OUD initiated on a 7‐day injectable XR‐BUP formulation across four EDs in the United States, using daily self‐reports and interval UDS. We secondarily explored how closely self‐reports matched UDS data during this period.

## 2 Methods

### 2.1 Setting and Participants

This is a secondary analysis of data from the nonrandomized, prospective single‐arm, open‐label, ancillary safety and feasibility study conducted in parallel with the larger ED‐INNOVATION randomized controlled effectiveness‐implementation trial [19]. The ancillary study was designed to determine the safety and feasibility of initiating CAM2038, a 7‐day injectable XR‐BUP formulation without a sublingual buprenorphine test dose, in patients with no to mild (COWS 0–7) opioid withdrawal. The results demonstrated that the XR‐BUP formulation was safe and well‐tolerated in patients presenting with COWS scores of 4–7 but not in those with COWS of 0–3. These findings allowed for the expansion of the inclusion criteria for the larger RCT to include patients with COWS scores of 4 and above [20].

The full protocol for the study has been previously published [20]. The study was conducted in 4 urban teaching hospitals' EDs in the Northeast, Mid‐Atlantic, and Pacific Coast geographic areas. For enrollment into the study, participants had to be 18 years of age or older, meet Diagnostic and Statistical Manual (DSM‐5) criteria for moderate‐to‐severe OUD, have a urine point‐of‐care toxicology test positive for opioids, have a Clinical Opiate Withdrawal Scale (COWS) [21] score less than 8 (denoting no‐mild withdrawal), and be able to speak English. Patients were excluded if urine toxicology was positive for methadone, they presented after an opioid overdose, were pregnant, actively suicidal, required opioids for pain, or enrolled in a program receiving medications for opioid use disorder in the past 14 days.

### 2.2 Baseline Visit

Participants who signed informed consent for study participation were administered a subcutaneous injection of 24 mg of CAM2038 and observed for 4 h prior to discharge. Relevant for this secondary analysis, all participants completed a baseline demographic survey, a 7‐day timeline follow‐back (TLFB) assessment of drug use, and a baseline point of care (POC) urine toxicology test in addition to several other measures.

### 2.3 Follow‐Up Visits and Assessments

Starting on postinjection day 1 and continuing daily through postinjection day 7, surveys were sent to each participant via text using Qualtrics. The survey included a visual analog scale to assess how much opioids were currently desired (craving), ranging from 0 to 100, and questions about the use of nonprescribed opioids or other drugs in the past 24 h (yes/no?). Drug use questions were not previously validated but are based on a validated timeline follow‐back methodology and provide a consistent, structured measure of daily use (Table S1) [22]. On postinjection day 7, participants were asked to provide a POC urine toxicology test and answer questions related to primary and secondary study outcomes.

### 2.4 Outcomes

Ancillary study outcomes have been previously reported [23]. For this secondary analysis, we report two outcomes that have not been previously described. We characterize the drug use patterns, as measured by daily self‐report and interval UDS in participants during the first week following initiation onto CAM2038. Second, we correlate baseline self‐reported drug use, as assessed by the TLFB, with baseline UDS results and self‐reported drug use during the first week of CAM2038 treatment, as assessed by daily surveys, with the day 7 UDS.

### 2.5 Statistical Analysis

Descriptive statistics (frequencies, measures of central tendency, and dispersion) were computed to characterize the data. Frequencies and percentages were used to describe drug use behavior.

For the analysis of drug use patterns as measured by TLFB and daily reports, only participants who completed at least four of seven potential surveys were included to ensure completeness and reliability in capturing meaningful trends over the week‐long follow‐up period. Inclusion of participants with highly limited data (e.g., only 1–3 days of reporting) could have disproportionately influenced results due to day‐to‐day variability in drug use and increased the risk of misclassification bias. For both baseline and Day 7 analyses, drug abstinence was recorded only if participants explicitly reported no drug use during the specified interval. For the analysis of drug use patterns as assessed by UDS, only participants with a baseline UDS and a UDS collected within the seven‐day window following the CAM2038 injection were included in the analysis. A generalized estimating equations (GEE) model with a repeated subject effect was used to calculate and test the average drug use of individual drugs pre vs. postinjection.

For the concordance analysis, the following parameters were used: Baseline concordance was demonstrated by comparing self‐reported drug use by TLFB and UDS, but only when both assessments were completed and the participant reported drug use within 3 days of study entry. Day 7 concordance was demonstrated by comparing self‐reported drug use by daily Qualtrics and the day 7 UDS. To increase the likelihood of reported drug use being identified by the UDS, analyses included only participants who provided self‐report data at least once within 3 days of providing their UDS, and whose UDS was collected within the seven‐day window following the CAM2038 injection. Concordance is reported as true positives (self‐report+, UDS+), true negatives (self‐report‐, UDS‐), false positives (self‐report+, UDS‐), and false negatives (self‐report‐, UDS+), sensitivity, and specificity. 95% confidence intervals for sensitivity and specificity were calculated using the Clopper–Pearson method. A higher percentage of true positives and true negatives demonstrates a greater degree of concordance, whereas lower percentages for false positives and false negatives demonstrate a greater degree of concordance. Concordance was tested using the McNemar test to account for within‐participant correlation.

Results were considered statistically significant at *p* < 0.05. Missing data were not imputed because of the relatively small sample size, which limits the reliability of imputation techniques. Given that this is an exploratory analysis, a sample size calculation and multiple testing adjustment were not performed. All analyses were conducted using SAS Version 9.4 (SAS Institute Inc., Cary, North Carolina). This study was approved by WIRB‐Copernicus Group (WCG).

## 3 Results

### 3.1 Participant Population Demographic Characteristics

Between July 30, 2020, and May 5, 2023, a total of 635 patients were evaluated for eligibility, of which 100 were enrolled [23]. Characteristics of the study population are shown in Table 1. Most (72%) of the participants were male, with 51% self‐identifying as white and 35% identifying as black or African American. Almost half (48%) of participants reported being unstably housed in the 12 months prior to enrollment, and 79% were publicly insured. The predominant route of opioid use was intranasal (45%), followed by injection (31%). Daily opioid use was high in this cohort, with the mean number of days of opioid use in the past week being 6 out of 7 days.

**TABLE 1:** Population Characteristics.

Population characteristics	Full population *N* = 100
Sex	
Male	72%
Age (Mean (SD))	36.5 (SD±8.70)
Race	
Asian	1%
Black or African American	35%
Multiracial	1%
White	51%
Other/unknown/refused	9%
Ethnicity	
Hispanic or Latino	13%
Unstable housing^1^ Past 12 Months	48%
Currently living in unstable housing^1^	36%
Insurance status	
None	6%
Public	79%
Private	13%
Missing/Other	1%
Route of opioid use	
Oral	13%
Nasal	45%
IV Injection	31%
Smoking	5%
Multiple Noninjection	4%
Other/NA/Unknown	2%
Number of days opioid used in the past 7‐days	
(Mean (SD))	6.0 (SD±1.79)

### 3.2 Drug Use Patterns as Measured by Timeline Followback and Daily Reports

**FIGURE 1:**
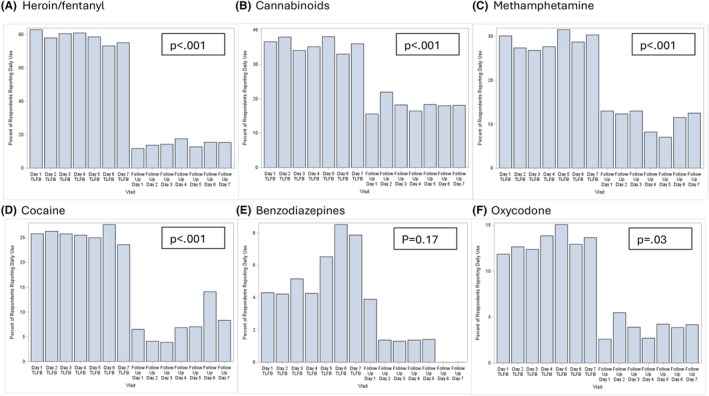
Self‐reported drug use in the 7 days prior to and following study enrollment.

All participants completed a baseline TLFB. Among these 81/100 completed at least 1 daily drug use survey, 79/100 completed at least four daily drug use surveys. Self‐reported drug use in the 7 days following treatment initiation decreased from baseline for all drugs (Figure 1). Among the 81 participants who completed at least 1 daily survey, most (98%, 79/81) reported at least 1 day with no opioid use and 63% (51/81) reported no opioid use for all 7 days.

### 3.3 Drug Use Patterns as Measured by Urine Drug Screens

**FIGURE 2:**
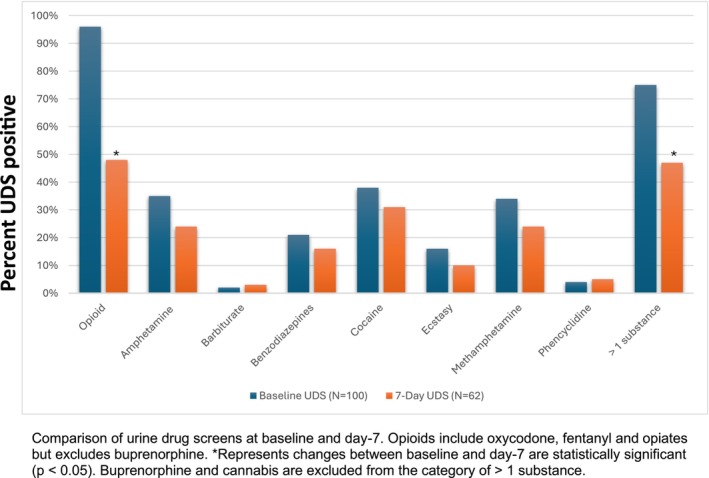
Drugs present in baseline and day–7 Urine Drug Screens.

All participants provided a baseline UDS, of which 62/100 provided a day 7 UDS. All participants had a baseline UDS with opioids (70% positive for fentanyl). There were 4 (4%) participants in whom the only opioid in the baseline UDS was buprenorphine. Excluding the study medication buprenorphine, drug use as measured by UDS declined from baseline to day 7 for all drugs with the exception of barbiturates and phencyclidine. These decreases were statistically significant for opioids and polydrug use (defined as more than 1 drug in the UDS excluding buprenorphine and cannabis) (Figure 2).

### 3.4 Concordance Between UDS Measured and Self‐Reported Drug Use

Concordance between self‐report and UDS at baseline and day 7 is shown in Table 2. Concordance between self‐reported drug use and UDS was moderate at baseline for cannabis (true positive 79%, true negative 95%, false positive 5%, false negative 21%), methamphetamine (true positive 77%, true negative 97%, false positive 3%, false negative 23%) and cocaine (true positive 71%, true negative 98%, false positive 2%, false negative 29%). Concordance between self‐reported drug use and UDS at day 7 was less for all drugs but still moderate for methamphetamine (true positive 73%, true negative 100%, false positive 0%, false negative 27%). Specificity was above 90% for all drugs except cannabis at both baseline and day 7. Sensitivities varied between 0% and 99% depending on the drug and time of measurement. Baseline sensitivities were higher than those at day 7 for all drugs, with the highest baseline sensitivities being found for cannabis (79%), methamphetamine (77%), and cocaine (71%), and the highest day 7 sensitivities being found for methamphetamine (73%) and cocaine (58%).

**TABLE 2:** Concordance between self‐report and UDS at baseline and day 7 follow‐up.

Drug	Timepoint	True positive (self‐reported +, UDS +)	False positive (Self‐reported +, UDS‐)	True negative (Self‐reported‐, UDS‐)	False negative (Self‐reported‐, UDS+)	Sensitivity (95% CI)	Specificity (95% CI)
Opioid	Baseline*	NA	NA	NA	NA	NA	NA
	Day 7	61% (19/31)	0% (0/30)	100% (30/30)	39% (12/31)	61% (42%–78%)	100.0% (88%–100%)
Cannabis	Baseline	79% (34/43)	5% (3/57)	95% (54/57)	21% (9/43)	79% (64%–90%)	95% (85%–99%)
	Day 7	48% (14/29)	6% (2/32)	94% (30/32)	52% (15/29)	48% (29%–67%)	94% (79%–99%)
Methamphetamine	Baseline	77% (26/34)	3% (2/66)	97% (64/66)	23% (8/34)	77% (59%–89%)	97% (89%–100%)
	Day 7	73% (11/15)	0% (0/46)	100% (46/46)	27% (4/15)	73% (45%–92%)	100% (92%–100%)
Cocaine	Baseline	71% (27/38)	2% (1/62)	98% (61/62)	29% (11/38)	71% (54%–85%)	98% (91%–100%)
	Day 7	58% (11/19)	0% (0/42)	100% (42/42)	42% (8/19)	58% (33%–80%)	100% (92%–100%)
Benzodiazepine	Baseline	24% (5/21)	1% (1/79)	99% (78/79)	76% (16/21)	24% (8%–47%)	99% (93%–100%)
	Day 7**	0% (0/10)	0% (0/51)	100% (51/51)	100% (10/10)	0% (0%–31%)	100% (93%–100%)

* Baseline opioid reporting is not applicable as all participants had to report opioid use and have a UDS containing opioids for study entry.

** There were no participants who completed a daily survey within three days of providing their day 7 UDS that contained benzodiazepines.

## 4 Discussion

This study provides the first comprehensive assessment of self‐reported and objectively measured drug use in the first week following ED‐initiated CAM2038. The primary finding from this secondary analysis was that the use of most drugs, as measured by both self‐reports and UDS, declined during the week following the initiation of XR‐BUP. While a reduction in opioid use was expected based on findings from other studies of XR‐BUP [24], the decrease in the use of nonopioid substances, including cocaine and methamphetamine, was an unexpected and important finding.

These results build upon existing evidence demonstrating the effectiveness of ED‐initiated buprenorphine in promoting treatment engagement and reducing opioid use. Given that the first weeks following an ED visit for an opioid‐related complaint are particularly high‐risk for morbidity and mortality [12], our findings suggest that initiating XR‐BUP in the ED may provide immediate benefits beyond reducing opioid use—potentially influencing polysubstance use patterns as well. Continued nonopioid drug use after treatment initiation is not unexpected, and our results align with prior research on office‐based buprenorphine initiation, which found that more than half of participants had urine specimens containing nonopioid substances, with cannabis, cocaine, and benzodiazepines being the most frequently detected in the first month of treatment initiation [25]. While one ED‐based buprenorphine initiation study demonstrated a reduction in nonprescribed opioid use in the 2 months following treatment initiation, it did not assess changes in the use of other substances [17].

The initial paper reporting results from this ancillary study trial focused on prespecified primary and secondary outcomes [19]. By contrast, the current analysis was not preplanned and therefore was not included in that original publication. We nevertheless believed it was important to examine immediate post‐discharge drug use patterns, as this represents a novel and clinically meaningful extension of the safety and feasibility findings of the trial. By suggesting that XR‐BUP initiation reduces drug use beyond opioids, our results amplify the implications of the original study and provide critical information for ED clinicians who must weigh short‐term overdose risk in post‐discharge care.

The clinical implications of these findings are significant. While previous studies have demonstrated that MOUD initiation reduces long‐term mortality [10, 13], our findings suggest that ED‐based initiation of XR‐BUP may contribute to short‐term overdose risk reduction by decreasing both opioid and non‐opioid substance use. The decreased detection of stimulants and benzodiazepines is particularly relevant given their impact on treatment retention and increasing overdose risk, especially in the context of fentanyl [26, 27, 28, 29].

A critical consideration in posttreatment care is the high prevalence of polysubstance use. While the reduction in nonopioid substance use observed in our study is encouraging, ongoing polysubstance use remains a concern. National trends indicate an increase in polysubstance use among individuals with OUD, which can complicate treatment engagement and increase overdose risk [15, 16]. Given that sustained polysubstance use has been associated with poorer retention in MOUD treatment [15], our findings highlight the need for comprehensive harm reduction strategies and tailored interventions beyond opioid‐focused treatment alone.

Another concern regarding MOUD initiation is the potential for risk compensation, a phenomenon where reductions in one risky behavior lead to increases in another. This concept has been well described in other public health interventions, such as HIV pre‐exposure prophylaxis [30]. Similarly, there is concern that reducing opioid use following buprenorphine initiation may be offset by increases in the use of other substances. The impact of MOUD on nonopioid substance use remains mixed in the literature, with some studies suggesting that patients receiving MOUD decrease their use of stimulants and cannabis, while others report an increase in nonopioid drug use following treatment initiation [31, 32, 33, 34]. Our study demonstrates that, at least in the short term, XR‐BUP initiation in the ED does not appear to drive increases in nonopioid drug use and may even contribute to reductions. However, continued monitoring of polysubstance use beyond the first week is needed to assess whether these reductions persist over time.

Another important aspect of this study was evaluating the concordance between self‐reported drug use and UDS results. In general, the high specificities at baseline and day 7 indicate that very few participants in this study who said they were using a drug did not have that drug detected in their UDS. Conversely, sensitivities were low to moderate for all drugs except opioids at baseline. This indicates that many participants who reported nonuse of a drug still had that drug detected in the UDS. This could be secondary to recall bias or social desirability effects, but could also be due to variable excretion times and concentrations of drugs measured in the UDS and protracted fentanyl clearance with heavy use [35]. We suggest interpreting this concordance data with caution, given that UDS results can be highly misleading when evaluating treatment success or failure [18]. For example, UDS results do not capture the frequency of use, and a positive result may look the same whether the drug was used once in a single day or multiple times across several days. If anything, these discrepancies underscore the importance of considering the use of subjective and objective measures when assessing substance use in ED‐based addiction treatment. While we do not believe a UDS should be required prior to initiating XR‐BUP in the ED, understanding the reliability of self‐reported drug use is particularly relevant to this setting, where rapid treatment decisions often rely on patient‐reported information.

## 5 Limitations

This study had several limitations, foremost being that it is a secondary analysis of a nonrandomized, single‐arm trial, limiting our ability to establish causal relationships. Furthermore, UDS results might not accurately reflect recent changes in drug use due to variable excretion times for each measured drug, and it is not possible to differentiate between concurrent and sequential polysubstance use. Despite this, concordance between self‐reported drug use and measured drug use tended to be moderate to high for most drugs at both baseline and follow‐up. However, there was substantial discordance in some cases, particularly false negatives, where participants denied use but had positive UDS results. These findings highlight the limitations of relying solely on self‐reports for research purposes and underscore the importance of interpreting such data with caution. Thirty‐eight participants did not provide a day 7 UDS or respond to daily drug surveys, resulting in missing data, which could have introduced bias if those lost to follow‐up were systematically different in their post‐treatment drug use patterns. It is also important to note that participants in this study agreed to receive an investigational medication under an IND protocol, which may indicate a higher level of motivation for treatment compared with the broader ED population with OUD. This potential selection bias should be considered when interpreting generalizability. Furthermore, while there was a relatively low rate of injection use (31%) and high prevalence of intranasal use (45%) observed in our cohort, this is consistent with recent national surveillance data showing declining injection and increasing smoking or insufflation of fentanyl and heroin [36, 37].

## 6 Conclusions

This study demonstrates that drug use, including both opioids and nonopioid substances, declines in the first week following ED‐initiated XR‐BUP treatment. These findings highlight the potential role of EDs in not only initiating MOUD but also reducing immediate post‐discharge overdose risk. The persistence of polysubstance use, however, underscores the need for continued harm reduction efforts and tailored interventions to address nonopioid substance use in this population.

## Author Contributions

Ethan Cowan had full access to all the data in the study and take responsibility for the integrity of the data and the accuracy of the data analysis. Gail D'Onofrio, David A. Fiellin, Jeanmarie Perrone and Ethan Cowan conceived the study and designed the trial. All authors contributed to data acquisition, analysis, and interpretation. Gail D'Onofrio, Ethan Cowan, Jeanmarie Perrone and David A. Fiellin drafted the manuscript, and all authors contributed substantially to its revision. Manali Phadke performed the statistical analysis. Gail D'Onofrio and David A. Fiellin obtained funding and supervised the study.

## Supporting information


**DATA S1:** acem70191‐sup‐0001‐Supinfo.zip.

## Data Availability

The data that support the findings of this study are available from the corresponding author upon reasonable request.
