# Supplementary material for: Drug Use After Emergency Department‐Initiated Injectable Buprenorphine: A Secondary Analysis of the ED‐INNOVATION Ancillary Safety and Feasibility Trial
Source: Acad Emerg Med. 2025 Nov 24;33(3):e70191. doi: 10.1111/acem.70191 (PMC12937049; doi:10.1111/acem.70191)
Supplement: Supplementary file 1 — DATA S1: acem70191‐sup‐0001‐Supinfo.zip. [file ACEM-33-0-s001.zip › acem70191-sup-0001-Supinfo1@Daily_Followup_Survey.pdf]

Daily Follow-Up Survey (DFS)

Dear Project **ED-INNOVATION participant**, thank you for completing the following questions!

1.Have you used any opioids not prescribed for you during the past 24 hours?

☐No ☐ Yes

a.If "Yes", what did you use?

☐Heroin/Fentanyl

☐Pain reliever or prescription pain medication (such as Percocet, Oxycodone, Vicodin)

☐Methadone (such as Dolophine, Methadose)

☐Other Opioids

i.You selected "Other Opioids". What did you use? ______________________________

2.Have you used any other drugs not prescribed for you during the past 24 hours?

☐No ☐ Yes

a.If "Yes", what did you use?

☐Marijuana (cannabis)

☐K2/Spice

☐Benzodiazepines (such as Valium, Ativan, Xanax, Librium, Rohypnol)

☐Methamphetamine (such as speed, crystal meth, ice)

☐Cocaine/Crack

☐Other drugs

i. You selected "Other drugs". What did you use? ________________________________

3.On a scale of 0 to 100 where 0 is definitely not and 100 is definitely so, how much you desire opioids at this moment?

"At this moment, I desire opioids:"

0 10 20 30 40 50 60 70 80 90 100

Definitely not Definitely so
